# Improving medical management of Eating Disorders in a community outpatient service

**DOI:** 10.1186/2050-2974-2-S1-P2

**Published:** 2014-11-24

**Authors:** Jenny Lawrence, Shannyn Lorkin

**Affiliations:** 1 ACT Health, Canberra, Australia

## 

Our community based Eating Disorder Program was set up to provide psychological and meal therapy support to consumers with eating disorders. Medical management of suffers remained with the GP.

We developed a growing doubt that medical management and communication between our service and GP's was less than excellent. We set about to investigate what was happening and improve this aspect of our service.

We collected baseline data on GP involvement taking a snap shot of current information. This data provided us the information we needed to proceed with change. We implemented a number of strategies to improve awareness of medical issues, client involvement with GP's, communication with GPs and record keeping.

Data collected 5 months later indicates improvements in these issues. Data will be presented and the strategies that we implemented discussed. Discussion will include the impact of the changes for clients, staff and also the future directions of our program. The project has assisted in giving us information that has changed our day to day practice and provided evidence to support future service development.

